# *"I could cry, the amount of shoes I can't get into": *A qualitative exploration of the factors that influence retail footwear selection in women with rheumatoid arthritis

**DOI:** 10.1186/1757-1146-4-21

**Published:** 2011-07-27

**Authors:** Serena Naidoo, Stephanie Anderson, Joanna Mills, Stephanie Parsons, Stephanie Breeden, Emma Bevan, Camilla Edwards, Simon Otter

**Affiliations:** 1 School of Health Professions, University of Brighton, Robert Dodd Building, 49 Darley Road, Eastbourne, BN20 7UR UK

## Abstract

**Background:**

Studies have reported that women with rheumatoid arthritis (RA) are not wearing NHS supplied therapeutic footwear; therefore it is likely they are wearing footwear sourced through retailers. Previous research gives limited information (largely associated with cosmesis) on people's perceptions on the relationships that exist between retail footwear, well-being and quality of life. This study aimed to explore the perceptions of women with RA regarding their choice of retail footwear and identify the factors influencing retail footwear selection.

**Methods:**

Eleven women with RA wearing normal retail footwear were recruited from an out-patient podiatry clinic in the south east of England. Semi-structured interviews were carried out and an interpretative phenomenological approach was adopted for data collection and transcript analysis.

**Results:**

Six key themes were revealed from the analysis: (1) the nature of foot complaints and deformities, (2) aesthetic appearance and design of footwear, (3) body image, (4) psychosocial aspects, (5) Perceptions of footwear and (6) the therapeutic value of retail shoes. These contributed to an overarching concept of loss of choice associated with retail footwear. In particular, the areas discussed most frequently throughout were themes (2), (3) and (4), which were notably more 'emotional' in nature.

**Conclusions:**

Limitations in retail footwear for these women have impacted on their individuality, linking significantly with their body image. The loss of choice in footwear as a consequence of the disease impacts negatively on emotions, wellbeing and was identified in reduced self-perceived quality of life.

## Background

Rheumatoid arthritis (RA) is a systemic, symmetrical inflammatory disease typically affecting several joints in the hands and feet [1]. This autoimmune disease causes synovial inflammation and destruction of joint architecture, leading to pain, loss of joint function, muscle atrophy from disuse and inability to participate in work and social activities [1-4]. These factors are often associated with disability and poor or reduced health-related quality of life [5,6]. The irreversible destructive changes within the foot often cause visible changes in foot structure, causing difficulty in finding safe and comfortable retail footwear [2,7]. A poorly fitting shoe may exacerbate foot complaints, induce further damage or cause falls due to reduced proprioceptive capacity, balance and postural reactions [8,9]. The impact of poorly fitting footwear may be under-estimated; indeed Williams and Bowden [10] reported that only 51% of people with rheumatic diseases were wearing suitable retail footwear. Those with inadequate footwear reported that their shoes contributed to or exacerbated their foot problems.

It is reported that the aims of foot care for patients with RA are to decrease pain, increase mobility and enhance quality of life [11,12]. However, achieving these aims can be difficult as those with RA often report frustration with everyday footwear citing difficulties with availability and choice [13], making goals associated with increasing activity unlikely, causing further emotional distress [14]. Many people with RA are prescribed therapeutic footwear but do not wear them, often due to poor fit or unacceptable cosmesis [15,16]; suggesting that the majority are therefore wearing retail footwear instead. However, the perception of retail footwear by people with RA has received little attention in the literature [17]. Furthermore, there is limited information concerning the relationships between footwear, well-being and quality of life (QOL). This study aimed to explore the perceptions of women with RA on their choice of footwear and identify the factors influencing footwear selection from within the retail sector.

## Methods

### Study Design

A modified interpretative phenomenological approach (IPA) was adopted whereby semi-structured interviews were used to explore a series of in-depth personal experiences of footwear by women with RA [18,19]. This type of focussed interview approach described by Merton et al [20] allows for a relaxed style but typically follows a set of questions derived from a protocol [21]. IPA is a philosophical approach, which acknowledges that the researcher applies interpretation to the data and that is influenced by the researchers' stance (clinician, woman, footwear wearer). This approach enabled researchers to focus on the exploration of participants' experiences, understandings, perceptions and views [22,23], in this case associated with retail footwear.

### Participants and Setting

The University of Brighton School Research Ethics and Governance Panel granted ethical approval. Potential participants were recruited from an out-patient podiatry clinic within the south east of England. Subjects were invited to participate on the basis that they met the criteria outlined in Table 1, established through reviewing their clinical records. Of the 11 women who were invited to participate, 8 agreed. One declined due to an impending planned hospital admission, another declined as her symptoms were in a state of flare at the time and the third declined as she was on holiday. All subjects provided informed written consent prior to participating. As an idiographic method, small sample sizes are considered normal in IPA [24,25].

**Table 1:** Inclusion Criteria

Female patients with a diagnosis of RA with the absence of additional systemic diseases such as diabetes, where therapeutic footwear may be required.
Age 18 or over

Able to converse fluently in English

Patients not prescribed or wearing therapeutic footwear e.g. orthopaedic shoes.

### Developmental Phase of the Study

Prior to undertaking this study all researchers were final year podiatry students who had participated in the same training in interview techniques. More than one interviewer was involved in generating data. This modified approach to IPA limited the potential risk of individual interviewers' epistemological perspective impacting on the research. Given that several researchers (SN, SA, SP, SB, JM, EB, CE) were to conduct at least one interview; the interview schedule (including how questions were asked) was jointly developed by the research team and based on findings from reviewing the literature around footwear provision. The key areas to explore included: footwear selection, footwear design and lifestyle choices. Interview questions were open-ended to allow for the introduction of new topics and the interview style was non-directive where possible [23]. The questions were tested on one individual in a developmental phase of the study to ensure this semi-structured protocol had face validity and generated data that met the aims of the study. Following the initial interview, the interview schedule was further refined (Additional file 1).

### Generation of data

Each interview took place within the out-patient clinic from which participants were recruited; a familiar setting for these individuals. The room used for interviewing differed from where treatment would normally occur to reduce patient anxiety, which may have affected verbal productivity [26]. No third parties were present during the interviews. Each interview lasted approximately one hour and light refreshments were served to participants. Informal conversation occurred before the commencement of interviews to put the participants at ease. All interviews were recorded; corresponding with the modified approach to IPA used in this study [27] (Additional file 2). Following an opening question enquiring generally about each subjects arthritis, questions and prompts were used to facilitate disclosure, maintain continuous conversation and to steer the participant back to the topics being discussed [19], enabling and facilitating the discussion between participant and interviewer.

### Data Analysis

Each interviewer transcribed their interview verbatim; pseudonyms were used to replace the names of participants both to ensure confidentiality while maintaining personality for each participant. The transcripts were distributed amongst the researchers to share the data collected; thus each interviewer read all seven transcribed interviews. Each transcript was read a number of times by each interviewer to identify recurring themes. These themes formed the basis of the results and corresponds with steps two and three of IPA [27] (Additional file 2). Themes were selected according to two criteria (i) prevalence of descriptions during the interviews and (ii) the articulacy and manner in which 'themes' assisted the participants' explanations [28]. This approach to analysis allowed for individual assessment of interpretation with minimal reflexivity; limiting the way personal experiences of researchers could influence data analysis [23,29]. To further ensure credibility and trustworthiness of data analysis, themes were discussed amongst the research group as a whole; creating a joint thematic framework for each interview [23] and therefore completing the final stage of IPA [27,30,31].

## Results

Demographic details of participants are outlined in Table 2 illustrating a varied range of participants each with different ages, lifestyles and duration of disease activity reflecting heterogeneity of those with RA. Six reoccurring themes were identified:

**Table 2:** Demographic Data for all participants

Participant	Age (Years)	Duration of disease (Years)	Employment Status	Marital Status
Lily	84	10	Retired	Married

Rose	36	1.5	Employed	Married with children

Violet	74	3	Retired	Married

Poppy	67	20	Retired	Married

Ivy	64	21	Volunteer	Widowed

Laurel	65	3	Retired	Married

Olive	77	34	Retired	Married with children

• the nature of foot complaints and deformities,

• body image and footwear,

• psychosocial aspects regarding RA and footwear,

• aesthetic appearance and design of footwear,

• the therapeutic value of retail shoes,

• perceptions of footwear.

With each theme, interview extracts have been selected as exemplars to represent the most expressive articulation of the corresponding theme [23].

### 1. The Nature of Foot Complaints and Foot Deformities

Physical foot deformities resulting from RA were reported by six interviewees. Notably, it was identified that the width of their feet had increased producing difficulties when trying to select retail footwear. Two respondents discussed their concerns with balance and falls due to their foot changes and having to wear larger shoes to accommodate deformities. Additionally, structural changes (e.g. bunions reported in two interviews) negatively impacted on footwear choice, which was described as 'limited' throughout. This also links with later results on the way in which women view their own feet because of these deformities.

*"You can see the shape of the joints and everything through the other side and that puts me off...I buy things that are one size too big so that your feet are comfortable." *Ivy, 64 years old, a volunteer, widowed.

Furthermore, all participants discussed foot pain, highlighting 'comfort' as a significant factor in footwear selection. The 'fit' of footwear due to pain highlighted the issues people with RA face on a daily basis affecting their footwear selection and impacting on their wellbeing and quality of life.

*"If I'm not comfortable then I might tend to trip...Comfort is the main thing otherwise I can't walk very well...I've had to throw out lots of shoes because they're not practical to wear." *Violet, 74 years old, retired, married.

Foot deformities coupled with foot pain were considerable limiting factors on everyday footwear selection.

### 2. Aesthetic Appearance and Design of Footwear

The aesthetic appearance and design of the footwear were described by all participants in various categories. Six reported colour as a key factor when selecting their shoes. Six identified materials (e.g. leather) as being important. Four preferred a cushioned rather than thin sole, which relates to comfort discussed above. Two participants reported difficulties experienced with the weight of retail footwear. All seven participants considered heel height as part of their selection process; either they would like to wear a heel or they would not be comfortable in a shoe that is too flat. This aspect also linked with appearance of shoes; for example, the type of heel e.g. 'slender' rather than 'stumpy' was highlighted. Five participants considered shoe fastenings both positively (these supported their feet), and negatively (these changed the aesthetic appearance of the footwear), which impacted on their collective thoughts of the aesthetic appearance of their shoe. For example, descriptions of pretty, dainty and attractive versus practical, bulky and ugly were commonly used relating to appearance of footwear. This latter issue in particular was closely related to body image.

*"Nothing dainty, nothing feminine looking; it's all big sturdy shoes with straps over...I would like to put a pair of heels on, but that's not going to happen." *Rose, 36 years old, employed, married, mother.

*"I used to be able to wear pretty shoes, now I'm stuck with lace-ups and flats...I hate the Velcro ones...I think it's ugly...I don't feel elegant." *Poppy, 67 years, retired, married.

Overall, the style of retail shoe now worn did not match the wishes and expectations of participants.

### 3. Body Image

Body image was identified in all seven of the interviews; creating the third theme. Femininity played a significant part in footwear selection. These subjects desired to commit to footwear that made them feel womanly, but lack of choice combined with their physical foot deformities had forced an altered body image to be adopted often leading to a change in social behaviour. This was also reflected through the frustration expressed of being restricted to certain clothing. This was mentioned particularly in relation to socialising and special occasions; burying the desired attire of dresses and skirts in the wardrobe along with the 'unworn' shoes.

*"I don't feel smart enough wearing flat shoes with a dress or a skirt...It's probably psychological but most ladies when they are wearing a skirt they like to have a little heel...It just makes you feel more dressed when you've got a little bit of a heel" *Laurel, 65 years old, retired, married.

*"If you go out in a skirt, you can't wear trainers..." *Olive, 77 years old, retired, married, mother.

Comparison of footwear between friends was mentioned in addition to the idea of being accepted within society. For example, these ladies wanted to wear shoes appropriate to the various seasons without having to feel visibly different from their peers. The youngest participant was particularly expressive about her body image and shoes and wanted to maintain her age and sexuality through these factors. She was very much aware of ageing before her time due to her footwear choices as seen in the quotes below.

*"It's quite annoying because I'm only 36 and I don't want to be wearing shoes that somebody about 70 should be wearing...I would love to wear something really girly but I just can't...a bit more feminine." *Rose, 36 years old, employed, married, mother.

*"My foot changes have necessitated a change in fashion...It's vanity because I'm a woman...I like to feel smart...I've just got to make the best of what I can find and dress accordingly" *Poppy, 67 years old, retired, married.

*"It does compliment an outfit to have a nice pair of shoes...First impressions people might look at your feet..." *Ivy, 64 years old, a volunteer, widowed.

Powerful emotions of shame, sadness and frustration were clearly identified by these women when speaking about their feet, footwear and body image.

### 4. Psychosocial Aspects

The psychosocial aspects recognised throughout the transcripts included lower self-esteem, how subjects felt they were seen by others, mood, depression, anger, frustration, social isolation, self-confidence, disappointment, bitterness, guilt and embarrassment.

*"People treat you differently with it [RA]...I don't go out very often...I look at my friends shoes and think 'I'd like those'...I feel a bit bitter to be honest." *Rose, 36 years old, employed, married, mother.

*"...you have to put up with what you've got...it can be frustrating...you just have to accept that." *Laurel, 65 years old, retired, married.

However, in contrast, acceptance of wearing the footwear and having these problems was also expressed which appeared on a number of occasions, coupled with some mechanisms of positivity and coping. For example, changing shoe style according to season.

*"I'm rather envious...In the winter everybody wears clumpy shoes, it doesn't seem to make much difference...But you know, come the summer...I can manage a sandal now and again in the summer and that boosts your confidence..." *Lily, 84 years old, retired, married.

These women recognised their negativity towards their disease and footwear, but were aware that this negativity was perhaps futile and therefore these positive and negative areas were reported in all seven of the interviews.

*"I put a pair on [of new shoes] and feel, and I think 'oh no, they're not going to be comfortable' and I have to just take them off, but never mind... [It makes me] cross and sad but you get used to it in the end..." *Lily, 84 years old, retired, married.

Moreover, being limited physically due to deformities and foot pain, but also from being unable to wear their desired shoes led to changes in social behaviour, which in turn impacted on the psychological well-being of these subjects.

### 5. Perceptions of Footwear

It was identified that four of the participants currently did not enjoy shoe shopping due to the limited choice available to them. Their perceptions of how their feet look in retail footwear in relation to their foot deformities and their reasons for selecting retail footwear (e.g. 'comfort') were key determinants that led to a negative perception of retail footwear.

*"I've found it such a trial finding shoes that are attractive and comfortable...I'm just frustrated and disappointed when I go around shops..." *Poppy, 67 years old, retired, married.

*"Before, [I had RA] I could choose something I would like and buy it but now I know that I can't do that because it's so uncomfortable..." *Poppy, 67 years old, retired, married.

Four participants also recognised a link between their negative perceptions of their footwear, fashion and ageing. Three subjects considered their perceptions to have changed with age. In contrast, two subjects had not changed their views since they had aged and found comfort to be more important than fashion.

*"...I think it changes with age anyway...When you get to my age, comfort is the most important thing...When people get to my age a lot of them have got other problems with their feet anyway so I think a lot of factors will kick in when you choose your footwear...I wish it was different" *Ivy, 64 years old, a volunteer, widowed.

The perception of footwear had changed for all participants as now, careful thought processes were considered something necessary in the maintenance of comfort.

### 6. The Therapeutic Value of Retail Shoes

Four participants relied on trainers for comfort or wore this type of footwear regularly, although one subject was rigidly against the use of such footwear due to their appearance.

*"When you've got trainers on you feel secure...my feet have got to last me a bit longer..." *Olive, 77 years old, retired, married, mother.

Five participants wore orthoses and discussed the impact of these on footwear selection. Feelings of guilt were often reported if orthoses were not worn because these devices did not fit into retail shoes; particularly as orthoses were found to be helpful in reducing pain. Therefore not wearing orthoses both increased foot pain and led to feelings of guilt.

*"I'm very limited, I can hardly get my feet into anything...I have insoles for my boots and trainers...I don't always find them comfortable, but I know I've got to wear them so I appreciate any help that I can get..." *Rose, 36 years old, employed, married, mother.

There remained considerable tensions between cosmetically acceptable retail shoes, the need to accommodate insoles to provide therapeutic benefit and overall comfort.

*"...the problem with having these sort of inserts *[orthoses] *is that I can't get them into any other shoes other than the shoes they were actually made for, so that's a major problem" *Laurel, 65 years old, retired, married.

In conclusion, the identification of these six themes illustrates the magnitude of feelings and thoughts considered by each participant. Their narratives highlight the key connections between loss of choice associated with retail footwear, body image and reduced self perceived quality of life.

## Discussion

Few previous studies have considered retail footwear, choosing to focus on hospital/orthopaedic shoes. In the current work participants revealed a number of reasons behind the selection of retail footwear in women with RA and six key areas of importance have been described. In particular, loss of choice due to aesthetic appearance and design of retail footwear, body image and psychosocial aspects surrounding footwear selection for people with RA were most frequently discussed. The inseparable combination of feet and footwear identified in this study reinforced the negative feelings of body image [32]. Previous work highlighted that therapeutic footwear can either emphasise or hide foot deformities in those affected, and well designed footwear can positively impact on well-being linking it directly with body image, perception of others, self esteem, mood and quality of life [32]. Our findings demonstrate for the first time similar findings for those with inflammatory arthritis who wear retail footwear. In parallel with previous work on therapeutic footwear [32,33], we found that all participants were concerned with loss of femininity and they considered themselves to be visibly different from their peers due to their limited choice of retail shoes, which created negative feelings and emotions about their footwear. Importantly, behaviour changes such as feeling restricted in clothing (because of limited footwear choices), thus feeling unable to attend social events created social isolation [33]. The detailed narratives revealed participants personal feelings and experiences associated with everyday shoes, which demonstrated how these psychosocial components magnified their suffering. The appearance and design of footwear was of considerable consequence for participants. For example, shoe fastenings emphasised the aesthetic appearance of shoes, in particular their unfeminine appearance. Equally, difficulties with the fit of orthoses/shoes were highlighted. Clinicians who provide foot orthoses need to be aware of the impact these devices can have on shoe fit and look to avoid further limiting of footwear choice.

A change in perceptions of footwear had evolved amongst participants and their ideas of an ideal shoe were now based on their current status of health rather than what they would choose to wear. Greco [34] emphasises that age is only one dimension when classifying consumers; for instance, when a person reaches the age of 60 it does not mean they are uninterested in clothing or fashion. The subjects in the current study had a mean age of 67 years, yet it was clearly identified that psychosocial factors, meaningful employment and fashion dilemmas were all related to their footwear choices [35]. Each participant admitted to owning far more pairs of shoes than they wore; the number actually worn ranged from 2-6 pairs. It was expressed that subjects felt 'reluctant' to throw them away, possibly as unworn shoes were hinged with previous memories.

Most studies indicate increased pain and loss of function as the main impact of RA on feet and therefore this impacts on footwear selection [32,36]. However, as with our findings other stressors such as appearance and well-being are also implicated [3]. The particular foot complaints identified by participants indicated their expertise in their own problems; they have strong control and a sense of ownership in their footwear selection process, which has been highlighted previously with both therapeutic and retail footwear [32,33,37]. Even if participants in the current study did not suffer with foot pain, they still encountered considerable difficulties with footwear selection. In women without systemic diseases; similar factors influencing footwear choice were identified including fashion, body image, and shoe size [38] highlighting that both shoe design and a complex inter-relationship of psychosocial factors surround choices around retail footwear. This suggests that women, regardless of a systemic disease, are affected by the same concerns because of the ontological associations between body image, fashion and shoes.

The use of a modified IPA in this study made it possible for the data to be interpreted on a deeper level; unique experiences of those with RA were uncovered facilitating a reflection on current practice. Equally, the involvement of several interviewers brought a broader perspective to the analysis. Moreover, all the interviews were carried out by female interviewers and Johnson [39] highlights that gender can be an important consideration in interviews, which in this case may have led to greater disclosure than may have occurred otherwise. That said, more than one interviewer may have led to difficulties in continuity between interviews or different personal philosophies that were not fully acknowledged. The use of a shared interview schedule and a reflexive approach to analysis limited these issues as far as possible. Video interviewing was a possible alternative and could have identified body language [19]. Additionally, it was considered that a friend or relative as a third party could have been present during the interviews to aid support to interviewees and to reduce possible stress [40]; but both of these latter techniques may have impacted on the trusted relationship and rapport built up between interviewers and interviewees.

In conclusion, it would appear that psychosocial factors, the design and appearance of shoes, body image, pain and deformities and the prescription of orthoses are all influencing factors that limit the choice and selection of retail footwear for women with RA. These factors impact on quality of life and clearly identify footwear as key personal and individual attributes to these women's lives and not just mere fashion accessories. This highlights the need for a patient-centred approach to the identification of suitable retail footwear as part of the clinical management of RA.

## Competing interests

The authors declare that they have no competing interests.

## Authors' contributions

All authors contributed equally to the conception and design of this study. SN, SA, JM, SP, SB, EB, CM collected and analysed data; SN & SO jointly submitted the manuscript, which all authors approved. SO was the principle investigator.

## Supplementary Material

Additional file 1 Interview Schedule: The modified interview schedule following the developmental phase of the study. Click here for file

Additional file 2 A Comparison of Aronson's Steps of IPA with the Researcher's Steps: A flow chart to illustrate the comparison between Aronson's Steps of IPA and the modified version of IPA used by the Researcher during data collection analysis. Click here for file
