# Supplementary material for: "I could cry, the amount of shoes I can't get into": A qualitative exploration of the factors that influence retail footwear selection in women with rheumatoid arthritis
Source: J Foot Ankle Res. 2011 Jul 27;4:21. doi: 10.1186/1757-1146-4-21 (PMC3166890; doi:10.1186/1757-1146-4-21)
Supplement: Additional file 1 — Interview Schedule: The modified interview schedule following the developmental phase of the study. [file 1757-1146-4-21-S1.DOC]

| **Questions** | **Prompts** |
| --- | --- |
| 1) Can you tell me about your arthritis? | - How long have you had RA? - What medication do you take? - Has your RA affected your feet? - How has it affected your feet? |
| 2) Can you tell me about the shoes you wear? | - How many pairs of shoes do you own? - How many can you actually wear comfortably? - Do you enjoy shoe shopping? - Do you find it difficult finding footwear which is comfortable? - Do you find your choice of footwear is limited? - Could you elaborate on these limitations? |
| 3) Do you find your shoes cause you discomfort? | - Do your shoes hurt you all the time or only after a while? - Do your shoes help you walk? - Would more comfortable shoes improve your ability to get out and about? |
| 4) What would your ideal footwear be and why? | - Can you describe the style of shoe? - Is colour important to you? - Is material important to you? - Does your ideal footwear change depending on the situation or where you are going e.g. somewhere special? |
| 5) How does your arthritis and the footwear you are able to wear affect your everyday life? | - Has the number of shoes you wear altered due to your arthritis? - Has there been a time when you have seen shoes you like but have been unable to wear? - May I ask you how that made you feel? - Do you feel your arthritis has affected your quality of life? |
