# Supplementary material for: "I could cry, the amount of shoes I can't get into": A qualitative exploration of the factors that influence retail footwear selection in women with rheumatoid arthritis
Source: J Foot Ankle Res. 2011 Jul 27;4:21. doi: 10.1186/1757-1146-4-21 (PMC3166890; doi:10.1186/1757-1146-4-21)
Supplement: Additional file 2 — A Comparison of Aronson's Steps of IPA with the Researcher's Steps: A flow chart to illustrate the comparison between Aronson's Steps of IPA and the modified version of IPA used by the Researcher during data collection analysis. [file 1757-1146-4-21-S2.DOC]

**Aronson’s Steps**  **Researcher’s Steps**

Step 1: Collect the data via audiotapes. From transcribed conversations, patterns of experiences can be listed

Step 1: Audio interviews collected and transcribed verbatim by each interviewer.

Step 2: Identify data that relates to classified patterns from step 1.

Step 2: Individual analysis of transcriptions to identify recurring patterns.

Step 3: Combine and catalogue related patterns into sub-themes: e.g. recurring activities, meanings, feelings.

Step 4: Discussion with group to classify patterns.

Step 5: Themes established

Step 4: Build a valid argument for choosing themes (i) read related literature, (ii) Develop theme statements.

Step 6: Exemplars from interviews used to enhance theme choices.

Step 3: Each interviewer read all 7 transcripts.
